# Amuvatinib Blocks SARS-CoV-2 Infection at the Entry Step of the Viral Life Cycle

**DOI:** 10.1128/spectrum.05105-22

**Published:** 2023-03-30

**Authors:** Trang T. X. Huynh, Thuy X. Pham, Gun-Hee Lee, Jae-Bong Lee, Sung-Geun Lee, Dongseob Tark, Yun-Sook Lim, Soon B. Hwang

**Affiliations:** a Laboratory of RNA Viral Diseases, Korea Zoonosis Research Institute, Jeonbuk National University, Iksan, South Korea; b Laboratory for Infectious Disease Prevention, Korea Zoonosis Research Institute, Jeonbuk National University, Iksan, South Korea; c Korea Zoonosis Research Institute, Jeonbuk National University, Iksan, South Korea; d Ilsong Institute of Life Science, Hallym University, Seoul, South Korea; University of Wisconsin-Madison

**Keywords:** SARS-CoV-2, COVID-19, amuvatinib, tyrosine kinase, therapeutic agent

## Abstract

Severe acute respiratory syndrome coronavirus 2 (SARS-CoV-2) is the causative agent of coronavirus disease 2019 (COVID-19). SARS-CoV-2 propagation is mediated by the protein interaction between viral proteins and host cells. Tyrosine kinase has been implicated in viral replication, and hence, it has become a target for developing antiviral drugs. We have previously reported that receptor tyrosine kinase inhibitor blocks the replication of hepatitis C virus (HCV). In the present study, we investigated two receptor tyrosine kinase-specific inhibitors, amuvatinib and imatinib, for their potential antiviral efficacies against SARS-CoV-2. Treatment with either amuvatinib or imatinib displays an effective inhibitory activity against SARS-CoV-2 propagation without an obvious cytopathic effect in Vero E6 cells. Notably, amuvatinib exerts a stronger antiviral activity than imatinib against SARS-CoV-2 infection. Amuvatinib blocks SARS-CoV-2 infection with a 50% effective concentration (EC_50_) value ranging from ~0.36 to 0.45 μM in Vero E6 cells. We further demonstrate that amuvatinib inhibits SARS-CoV-2 propagation in human lung Calu-3 cells. Using pseudoparticle infection assay, we verify that amuvatinib blocks SARS-CoV-2 at the entry step of the viral life cycle. More specifically, amuvatinib inhibits SARS-CoV-2 infection at the binding-attachment step. Moreover, amuvatinib exhibits highly efficient antiviral activity against emerging SARS-CoV-2 variants. Importantly, we demonstrate that amuvatinib inhibits SARS-CoV-2 infection by blocking ACE2 cleavage. Taken together, our data suggest that amuvatinib may provide a potential therapeutic agent for the treatment of COVID-19.

**IMPORTANCE** Tyrosine kinase has been implicated in viral replication and has become an antiviral drug target. Here, we chose two well-known receptor tyrosine kinase inhibitors, amuvatinib and imatinib, and evaluated their drug potencies against SARS-CoV-2. Surprisingly, amuvatinib displays a stronger antiviral activity than imatinib against SARS-CoV-2. Amuvatinib blocks SARS-CoV-2 infection by inhibiting ACE2 cleavage and the subsequent soluble ACE2 receptor. All these data suggest that amuvatinib may be a potential therapeutic agent in SARS-CoV-2 prevention for those experiencing vaccine breakthroughs.

## INTRODUCTION

Severe acute respiratory syndrome coronavirus 2 (SARS-CoV-2) belongs to the *Coronaviridae* family that includes Middle East respiratory syndrome coronavirus (MERS-CoV) and SARS-CoV-1 (1). SARS-CoV-2 is an enveloped, single-stranded, positive-sense RNA virus with a large size genome of ~30 kb. Two nonstructural proteins, open reading frame 1a (ORF1a) and ORF1b, are directly translated from the genomic RNA and then further cleaved by two viral proteases into 16 nonstructural proteins. Discontinuous subgenomic RNAs (sgRNAs) encode four structural (S, E, M, and N) proteins and six accessory (3a, 6, 7a, 7b, 8, and 9b) proteins in the infected cells (2). SARS-CoV-2 enters host cells by using the spike (S) protein. The S protein is a homotrimeric glycoprotein with three receptor-binding S1 proteins and membrane fusion S2. S1 contains a receptor-binding domain that recognizes angiotensin-converting enzyme 2 (ACE2) as its receptor. The S protein is proteolytically activated at the S1-S2 boundary, and S1 is dissociated from S2. The S protein cleavage and activation are regulated by transmembrane protease serine 2 (TMPRSS2) (3–5). ACE2 has been identified as a cellular receptor for both SARS-CoV and SARS-CoV-2. It has been reported that the S protein of SARS-CoV-2 binds ACE2 with higher affinity than that of SARS-CoV (6). ACE2 is a transmembrane protease and can be cleaved by TMPRSS2. ACE2 cleavage produces soluble ACE2 (sACE2), resulting in loss of the membrane-bound form (7). sACE2 binds to SARS-CoV-2 as a receptor and then mediates viral entry into host cells (8, 9). The cleavage of ACE, also called ACE shedding, from the cell surface plays an important role in SARS-CoV-2 infection. TMPRSS2 promotes SARS-CoV entry either by promoting virus uptake via ACE2 cleavage or by activating the S protein for membrane fusion (10). In SARS-CoV-2, ACE2 shedding by TMPRSS2 is required for TMPRSS2-mediated enhancement of fusion in the absence of S1-S2 priming (11). S is the major protein that determines the specificity of the virus for cell types and becomes the main target for neutralizing antibodies against SARS-CoV-2 infection.

SARS-CoV-2 is the causative agent of coronavirus disease 2019 (COVID-19) and has caused nearly 750 million cases of infections and over 6.8 million deaths worldwide as of February 2023 (12). SARS-CoV-2 exploits host cellular machinery for all steps of its life cycle, and thus, any drug that can disrupt the viral life cycle could be a potential therapeutic agent to treat COVID-19 patients. Targeting the host protein has some advantages in high genetic barriers to resistance.

Receptor tyrosine kinases (RTKs) mediate signaling cascade in response to external and internal stimuli and play important roles in diverse biological processes, including cell proliferation, differentiation, metabolism, and apoptosis (13). Alterations of RTKs are involved in tumorigenesis and progression (14–18) and thus become major targets for drug discovery. Tyrosine kinase inhibitors (TKIs) block the activation of downstream signaling pathways, and TKIs have been used to treat cancer patients (19). Recent studies show that TKIs have antiviral activities against various viruses, including HIV (20), Zika (21), Ebola virus (22), influenza A virus (23), and SARS-CoV-2 (24). Imatinib, one of the examples of highly potent inhibitors of RTKs, has been reported to inhibit SARS-CoV-1 and MERS-CoV infection by inhibiting the fusion of the virions at the endosomal membrane. Amuvatinib is also a multitargeted tyrosine kinase inhibitor that suppresses c-kit, platelet-derived growth factor receptor (PDGFR), c-MET, and FLT3 (25). Amuvatinib has been shown to exert antiviral activities against Zika virus (26) and MERS-CoV (27). In the present study, we investigated whether amuvatinib could block SARS-CoV-2 propagation. Using spike protein as pseudoparticles (SARS-CoV-2pp), we show that amuvatinib, but not imatinib, inhibits SARS-CoV-2 infection in Vero E6, Calu-3, and HEK293T cells. We further demonstrate that amuvatinib efficiently blocks SARS-CoV-2 in the early stage of viral infection.

## RESULTS

### Evaluation of tyrosine kinase inhibitors as a potential drug against SARS-CoV-2.

**FIG 1:**
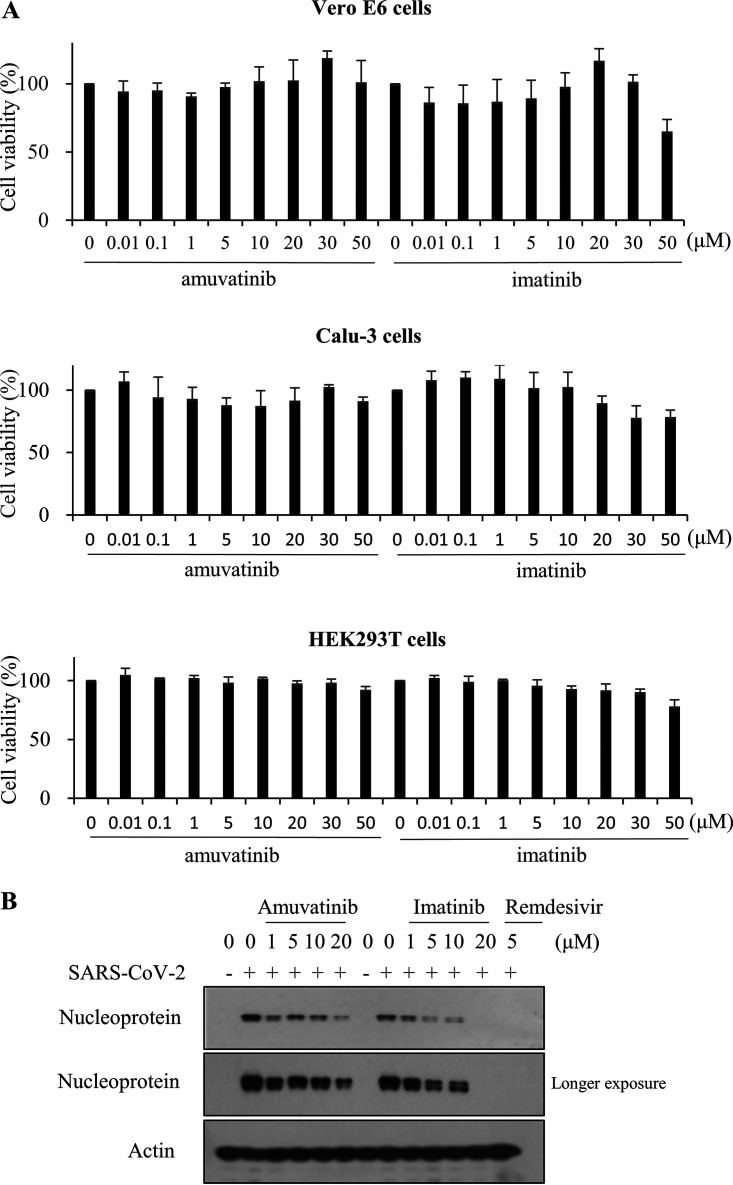
Amuvatinib inhibits SARS-CoV-2 infection. (A) Vero E6, Calu-3, and HEK293T cells were treated with various concentrations of amuvatinib or imatinib. At 48 h after treatment, cell viability was determined by WST assay using water-soluble tetrazolium salt. Data represent averages from triplicate experiments. (B) Vero E6 cells were either mock infected or infected with SARS-CoV-2 (MOI = 0.01) for 1 h in the absence or presence of the indicated chemicals and further cultured in media containing various concentrations of either amuvatinib or imatinib. At day 2 postinfection, SARS-CoV-2 protein levels were determined by immunoblot analysis using the indicated antibodies. Immunoblots were performed in triplicates. Remdesivir was used as a positive control. All experiments were performed in a BSL-3 facility.

We first examined the cytopathic effect (CPE) of SARS-CoV-2 in Vero E6 cells. Cells were either mock infected or infected with SARS-CoV-2, and then viral cytopathogenicity was monitored by light microscopy. At 36 h postinfection, we clearly observed SARS-CoV-2-induced CPE in Vero E6 cells (see Fig. S1A in the supplemental material). We showed that 5 μM remdesivir completely blocked SARS-CoV-2-induced CPE. Remdesivir is used as a positive control since it is an FDA-approved SARS-CoV-2 drug. To test the effects of tyrosine kinase inhibitors, amuvatinib and imatinib, on SARS-CoV-2 infection and replication, Vero E6 cells were inoculated with a mixture of SARS-CoV-2 (multiplicity of infection [MOI] = 0.01) and amuvatinib or imatinib for 1 h. At 48 h postinfection, cell morphology was examined under a light microscope. As shown in Fig. S1B, SARS-CoV-2-induced CPE was inhibited by 10 μM of either amuvatinib or imatinib. Strikingly, CPE was markedly blocked by both chemicals at the concentration of 20 μM. It was noteworthy that treatment of 50 μM of each chemical displayed no effect on the viability of Vero E6, Calu-3, and HEK293T cells (Fig. 1A), indicating that these drugs potently inhibited SARS-CoV-2 propagation within the nontoxic concentration range. We further demonstrated that both chemicals decreased SARS-CoV-2 protein levels in a dose-dependent manner (Fig. 1B). Data from triplicate immunoblots are summarized as a plotted graph in Fig. S2.

### Characterization of amuvatinib as an anti-SARS-CoV-2 agent.

**FIG 2:**
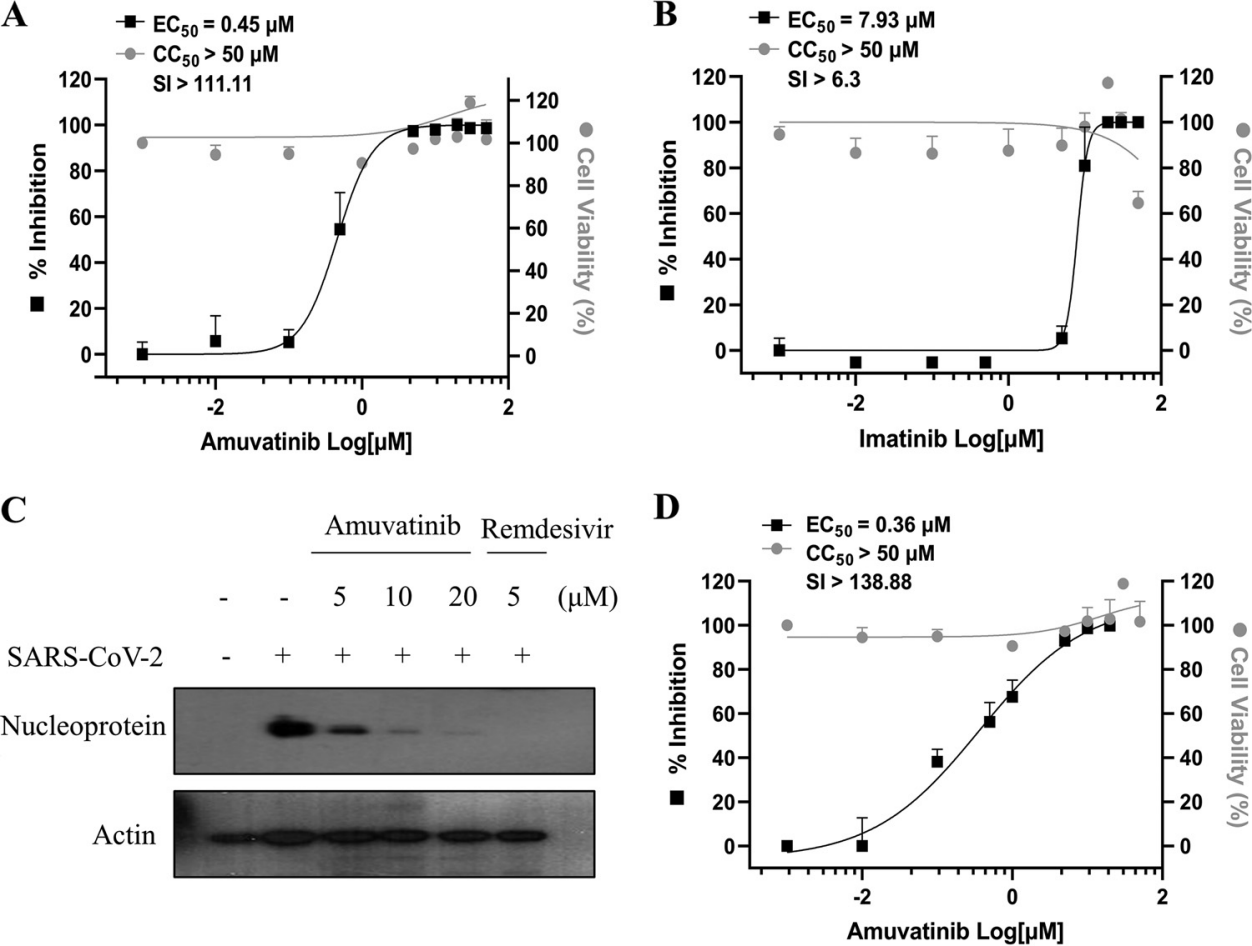
Confirmation of anti-SARS-CoV-2 activity of amuvatinib. (A, B) Vero E6 cells were either mock infected or infected with SARS-CoV-2 (MOI = 0.01) in the presence of various concentrations of either amuvatinib (A) or imatinib (B) for 1 h. Cells were fed with fresh media containing the indicated chemicals. At 24 h after chemical treatment, the TCID_50_ value was determined from the SARS-CoV-2-containing supernatant. The EC_50_ value was determined from the value of TCID_50_. Data represent averages from triplicate experiments. (C) Vero E6 cells were either mock infected or infected with SARS-CoV-2 (MOI = 0.01) in the absence or presence of various concentrations of amuvatinib for 1 h. The SARS-CoV-2-infected cells were further cultured in fresh media containing amuvatinib. At 24 h postinfection, SARS-CoV-2 nucleoprotein levels were determined by an immunoblot assay. Immunoblot analysis was performed in triplicates. (D) Vero E6 cells were treated as described in panel C. At 24 h postinfection, total RNA was extracted, and the EC_50_ value was determined by qRT-PCR. Data represent averages from triplicate experiments. Dose-response curves for EC_50_ and CC_50_ values were determined by nonlinear regression analysis using GraphPad Prism 9. Compound efficacy was determined by the selectivity index (SI). SI, ratio between cytotoxicity (CC_50_) and antiviral activity (EC_50_).

Since the effect of imatinib on SARS-CoV-2 infection and replication has been previously reported (28), we chose amuvatinib to explore the possible drug candidate for SARS-CoV-2 in this study. To verify the antiviral effect of amuvatinib against SARS-CoV-2, we determined the 50% tissue culture infective dose (TCID_50_) and compared anti-SARS-CoV-2 activity between amuvatinib and imatinib in Vero E6 cells as described in Materials and Methods. As shown in Fig. 2, the 50% effective concentration (EC_50_) of amuvatinib is 0.45 μM (Fig. 2A), whereas the EC_50_ of imatinib is 7.93 μM (Fig. 2B), indicating that amuvatinib exhibits higher anti-SARS-CoV-2 activity than imatinib in Vero E6 cells. To further confirm the anti-SARS-CoV-2 activity of amuvatinib, we analyzed viral protein expression in amuvatinib-treated cells. Vero E6 cells were inoculated for 1 h with a mixture of SARS-CoV-2 (MOI = 0.01) and amuvatinib, and then media were replaced with fresh media containing chemicals. At 24 h postinfection, the viral protein level was analyzed by an immunoblot assay. Figure 2C showed that SARS-CoV-2 protein levels decreased by amuvatinib in a dose-dependent manner. Data from triplicate immunoblots are summarized as a plotted graph in Fig. S3. Remdesivir was used as a positive control. Using quantitative real-time PCR (qRT-PCR) data, we further confirmed that amuvatinib blocked SARS-CoV-2 infection in Vero E6 cells, with an EC_50_ value of 0.36 μM (Fig. 2D). Considering that the 50% inhibitory concentration (IC_50_) value of remdesivir is 0.098 μM (29), the anti-SARS-CoV-2 activity of amuvatinib (EC_50_ = 0.36 μM) is assumed to be highly effective. These results indicate that amuvatinib may represent a potent blocking agent against SARS-CoV-2 propagation.

### Amuvatinib inhibits SARS-CoV-2 propagation in human bronchial epithelial Calu-3 cells.

**FIG 3:**
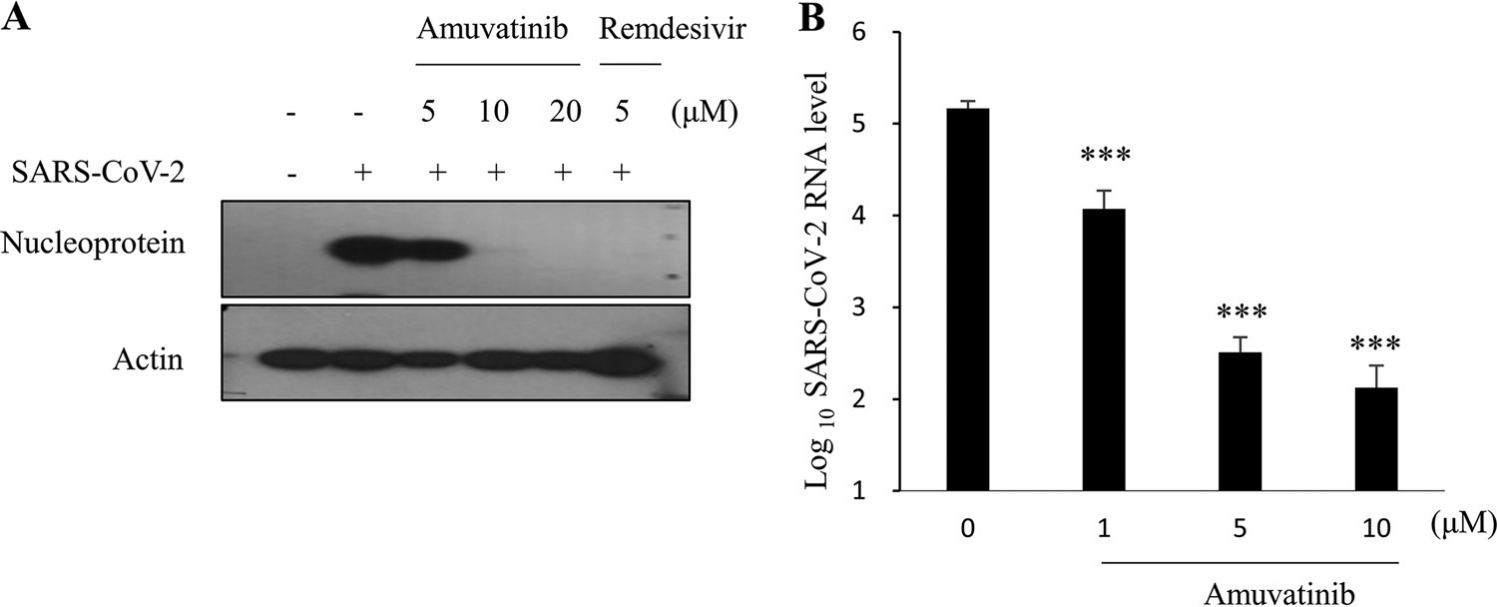
Amuvatinib blocks SARS-CoV-2 propagation in human lung Calu-3 cells. (A) Calu-3 cells were either mock infected or infected with SARS-CoV-2 (MOI = 0.1) for 1 h in the presence of various concentrations of amuvatinib. At 48 h postinfection, cells were harvested to determine protein levels by an immunoblot assay using the indicated antibodies. Immunoblot analysis was performed in triplicates. (B) Calu-3 cells were treated as described in panel A. At 24 h postinfection, total RNA was extracted, and then SARS-CoV-2 RNA levels were determined by qRT-PCR. Data represent averages from triplicate experiments. ***, *P* < 0.001.

Calu-3 is a human lung cancer cell line that has been used to confirm the anti-SARS-CoV-2 effect of a testing drug. Here, we investigated the antiviral effect of amuvatinib on SARS-CoV-2 propagation by inoculating Calu-3 cells with SARS-CoV-2 at an MOI of 0.1 in the presence of various concentrations of amuvatinib. As shown in Fig. 3A, amuvatinib decreased SARS-CoV-2 nucleoprotein levels in a dose-dependent manner. Notably, 10 μM amuvatinib decreased viral nucleoprotein to a nearly undetectable level. Data from triplicate immunoblots are summarized as a plotted graph in Fig. S4. As expected, SARS-CoV-2 RNA levels were also significantly decreased by amuvatinib (Fig. 3B). These results verify that amuvatinib significantly blocks SARS-CoV-2 propagation in human lung cells.

### Amuvatinib blocks SARS-CoV-2 propagation at the binding-attachment stage.

**FIG 4:**
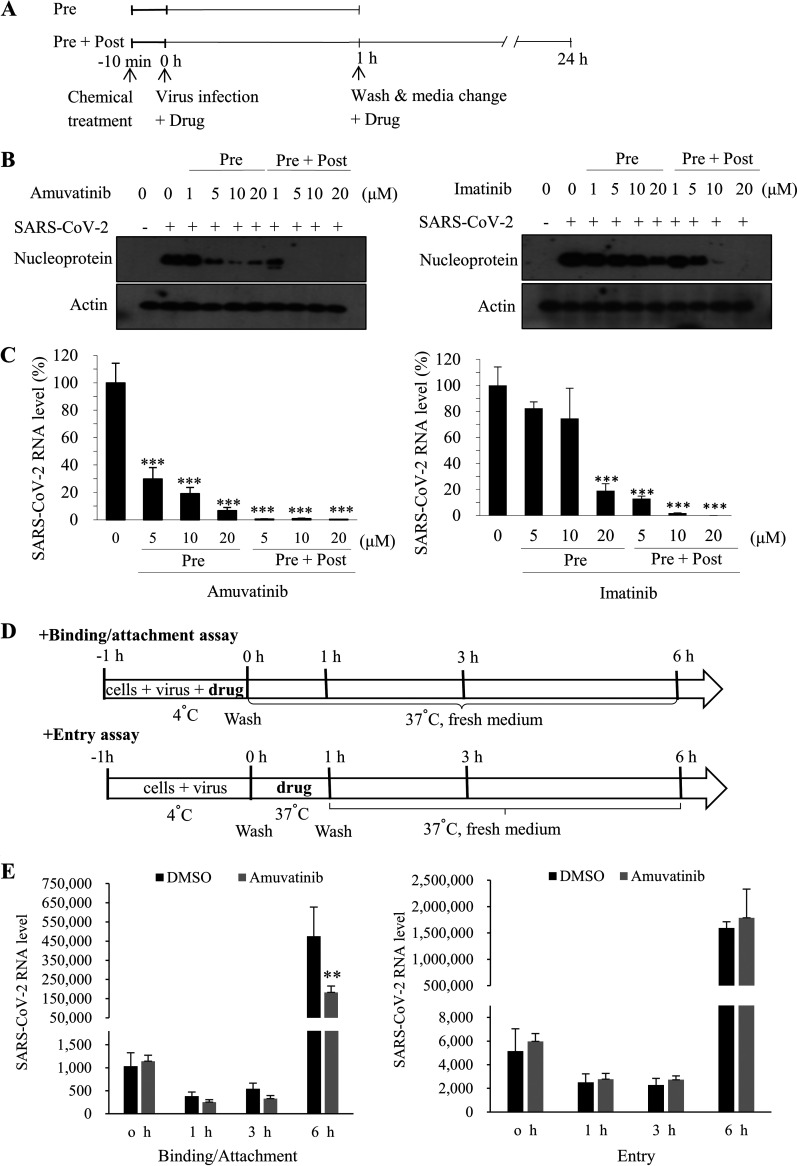
Amuvatinib blocks SARS-CoV-2 at the binding-attachment stage. (A) Schematic illustration of the experimental design. (B) Vero E6 cells were pretreated with various concentrations of either amuvatinib or imatinib for 10 min and then inoculated with SARS-CoV-2 (MOI = 0.01) for 1 h in the presence of each chemical. The culture medium was replaced with fresh medium in the absence of chemicals (pretreatment [Pre]), or Vero E6 cells were pretreated with various concentrations of either amuvatinib or imatinib for 10 min and then inoculated with SARS-CoV-2 for 1 h in the presence of each chemical. The culture medium was replaced with fresh medium in the presence of various dosages of chemicals (pre- plus posttreatment [Pre + Post]). At 24 h postinfection, SARS-CoV-2 protein levels were determined by immunoblot analysis. All immunoblot experiments were performed in triplicates. (C) Total RNA was extracted from Vero E6 cells, and SARS-CoV-2 RNA levels in cells treated with either amuvatinib or imatinib were determined by qRT-PCR. Data represent averages from triplicate experiments. (D) Schematic illustration of the experimental design. (E) For the binding-attachment assay, Vero E6 cells were incubated with SARS-CoV-2 (MOI = 1) at 4°C for 1 h in the presence of 20 μM amuvatinib. The cells were washed with PBS, and then the temperature was shifted to 37°C. At the indicated time points, total RNA was extracted, and then SARS-CoV-2 RNA levels were determined by qRT-PCR. For the entry assay, Vero E6 cells were incubated with SARS-CoV-2 (MOI = 1) at 4°C for 1 h in the absence of amuvatinib. The cells were washed with PBS, and then the temperature was shifted to 37°C in the presence of 20 μM amuvatinib. At 1 h after chemical treatment, the cells were washed twice with PBS. At the indicated time points, SARS-CoV-2 RNA levels were determined by qRT-PCR. Data represent averages from triplicate experiments. Data were analyzed by two-way ANOVA with Bonferroni multiple comparisons. **, *P* < 0.01; ***, *P* < 0.001.

To determine which step of the life cycle of SARS-CoV-2 is affected by amuvatinib, Vero E6 cells were treated with either amuvatinib or imatinib in two different ways, pretreatment or pre- and posttreatment (pre-post) (Fig. 4A). The cells were preincubated with either amuvatinib or imatinib for 10 min and then inoculated with SARS-CoV-2 for 1 h in the presence of various concentrations of each chemical. The culture medium was replaced with fresh medium in the absence (pretreatment) or presence (pre-post) of various concentrations of each chemical for 24 h. Figure 4B shows that SARS-CoV-2 nucleoprotein levels markedly decreased by 5 μM amuvatinib under the pretreatment condition (left). Meanwhile, viral protein levels slightly decreased by imatinib under the pretreatment condition (Fig. 4B, right). We found that antiviral activity was higher at pre-post than the pretreatment-only condition in cells treated with either amuvatinib or imatinib. It was noteworthy that a low dosage of amuvatinib, but not imatinib, efficiently blocked SARS-CoV-2 propagation (Fig. 4B, left versus right panels). Next, we also assessed SARS-CoV-2 RNA levels. Total RNA was isolated from SARS-CoV-2-infected Vero E6 cells treated with either amuvatinib or imatinib as described in Fig. 4A, and then viral RNA levels were determined by qRT-PCR. As shown in Fig. 4C, amuvatinib significantly decreased SARS-CoV-2 RNA levels under both conditions in a dose-dependent manner (left), whereas SARS-CoV-2 RNA levels were significantly decreased by imatinib in a dose-dependent manner only under the pre-post condition but not the pretreatment condition (Fig. 4C, right). These data indicate that amuvatinib, but not imatinib, efficiently inhibits SARS-CoV-2 propagation at the infection step of the life cycle. To precisely determine which step of the SARS-CoV-2 life cycle was targeted by amuvatinib, we divided the infection step into binding-attachment and entry steps, as shown in Fig. 4D. For the binding-attachment assay, Vero E6 cells were inoculated with SARS-CoV-2 (MOI = 1) in the presence of 20 μM amuvatinib at 4°C for 1 h and washed in phosphate-buffered saline (PBS), and then the temperature was shifted to 37°C. For the entry assay, Vero E6 cells were inoculated with SARS-CoV-2 (MOI = 1) in the absence of amuvatinib at 4°C for 1 h, and then temperature was shifted to 37°C in the presence of inhibitor. At 1 h post-drug treatment, cells were washed in PBS. At the indicated time points, SARS-CoV-2 RNA levels were determined by qRT-PCR. We showed that SARS-CoV-2 RNA levels abruptly and significantly increased at 6 h in each experiment (Fig. 4E). Of note, amuvatinib significantly decreased SARS-CoV-2 RNA levels at the binding-attachment step but not at the entry step. All these data indicate that amuvatinib specifically inhibits SARS-CoV-2 infection at the binding-attachment step of the virus life cycle.

### Amuvatinib inhibits SARS-CoV-2 variant propagation in human lung cells.

**FIG 5:**
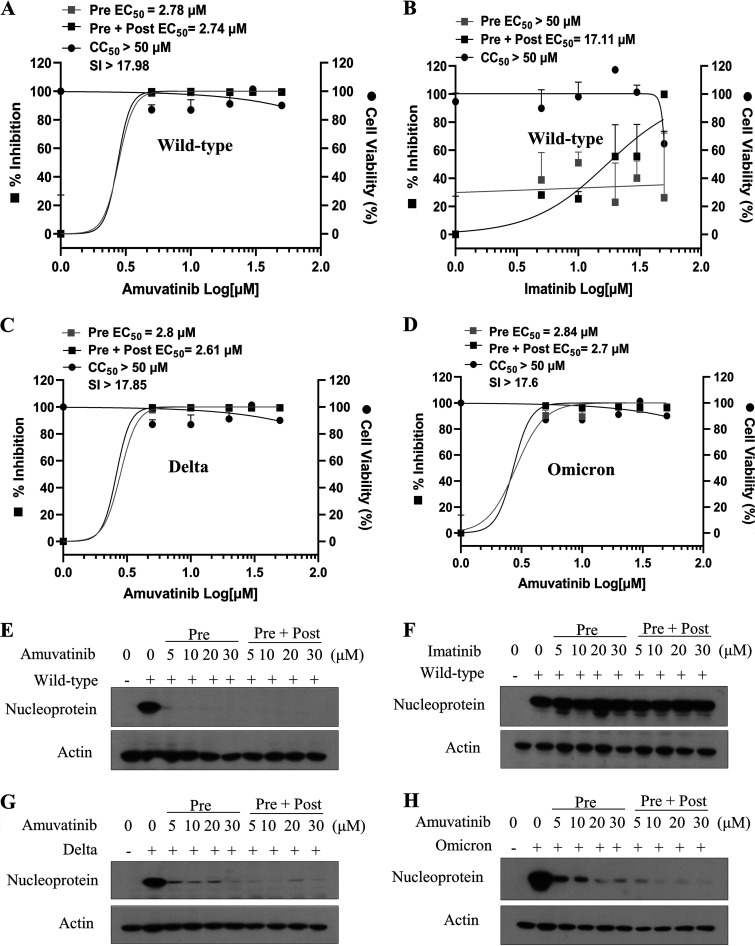
Amuvatinib inhibits SARS-CoV-2 variant propagation at the early step of infection in human lung cells. (A to D) Calu-3 cells were infected with either wild-type SARS-CoV-2 (MOI = 0.1) (A, B), Delta (MOI = 1) (C), or Omicron (MOI = 2) (D) and treated with either amuvatinib or imatinib as described in Fig. 4A and B. The EC_50_ value was determined as described in Fig. 2. Data represent averages from triplicate experiments. Dose-response curves for EC_50_ and CC_50_ values were determined by nonlinear regression analysis using GraphPad Prism 9. Compound efficacy was determined by SI (CC_50_/EC_50_). (E to H) Calu-3 cells were treated as described in panels A to D. At 48 h postinfection, viral protein expression levels were determined by immunoblot assay using the indicated antibodies. Immunoblot analysis was performed in triplicates.

To verify whether amuvatinib blocks SARS-CoV-2 propagation at the binding-attachment step in human lung cells, Calu-3 cells were treated as described in Fig. 4A. At 48 h postinfection, EC_50_ values were determined. Notably, EC_50_ values of pretreatment (2.78 μM) and pre-post (2.74 μM) conditions were comparable in amuvatinib-treated wild-type SARS-CoV-2-infected Calu-3 cells (Fig. 5A). However, the EC_50_ value of pretreatment (>50 μM) in imatinib-treated SARS-CoV-2-infected cells was profoundly higher than that of pre-post (17.11 μM). We next investigated whether amuvatinib blocked viral propagation at the binding-attachment step in SARS-CoV-2 variants. As shown in Fig. 5, EC_50_ values of pretreatment and pre-post in amuvatinib-treated Delta (Fig. 5C) and Omicron (Fig. 5D) variants of SARS-CoV-2-infected Calu-3 cells were similar to that of wild-type SARS-CoV-2. To further analyze the effect of amuvatinib on SARS-CoV-2 infection between the binding-attachment and entry steps, we analyzed viral protein expression levels in pre-post conditions. We verified that amuvatinib, but not imatinib, potently inhibited SARS-CoV-2 propagation as determined by viral protein expression levels (Fig. 5E versus Fig. 5F). We further showed that amuvatinib blocked viral propagation at the binding-attachment step in both Delta (Fig. 5G) and Omicron stealth (Fig. 5H) variant-infected Calu-3 cells. These data confirm that amuvatinib displays pan-genotypic inhibition of SARS-CoV-2 in human lung cells.

### Amuvatinib inhibits SARS-CoV-2 pseudovirus infection in various cell lines.

**FIG 6:**
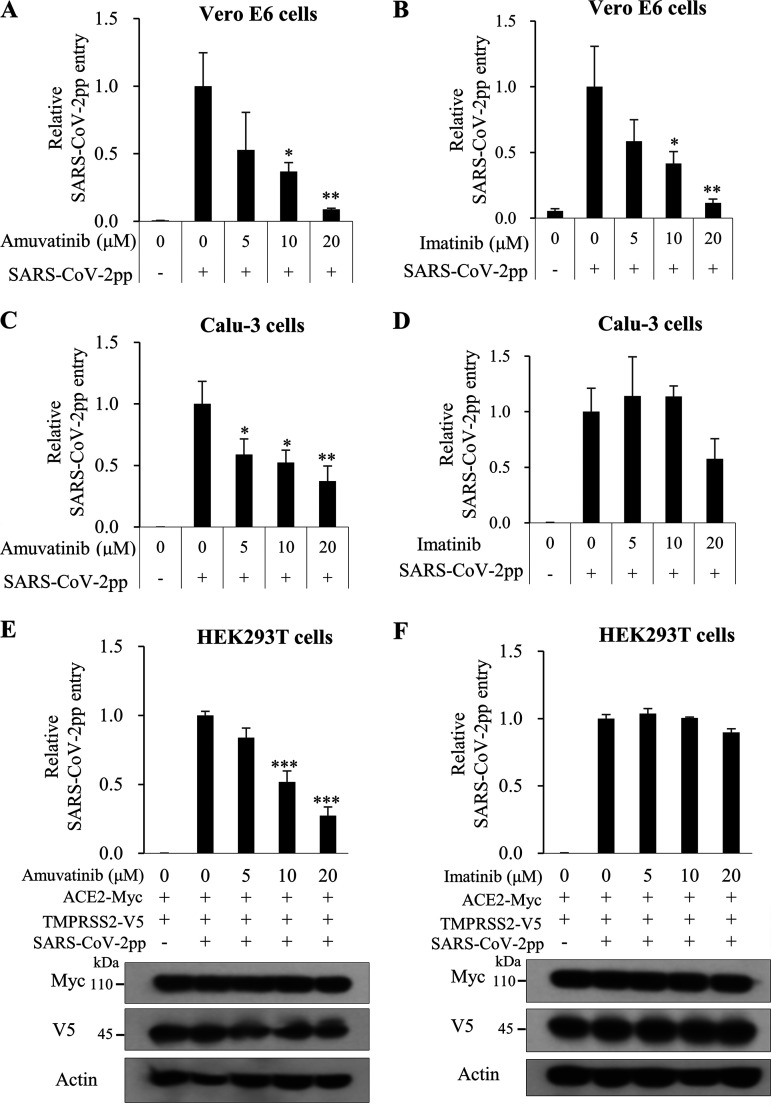
Amuvatinib inhibits SARS-CoV-2 pseudovirus infection in various cell lines. (A and B) Vero E6 cells were infected with SARS-CoV-2pp in the absence or presence of various concentrations of either amuvatinib (A) or imatinib (B) for 1 h, and then culture media were replaced with fresh media without chemicals. At 36 h postinfection, the cells were harvested, and viral infection was determined by luciferase assay. (C and D) Calu-3 cells were infected with SARS-CoV-2pp, and viral entry was determined as described in panels A and B. (E and F, Top) HEK293T cells were either transfected with empty vector, Myc-tagged ACE2, or V5-tagged TMPRSS2 plasmid or cotransfected with Myc-tagged ACE2 and V5-tagged TMPRSS2 for 20 h and then infected with SARS-CoV-2pp in the absence or presence of various concentrations of either amuvatinib (E) or imatinib (F) for 1 h, and then culture media were replaced with fresh media without chemicals. At 36 h postinfection, viral infection was determined by luciferase assay. (E and F, Bottom) Protein expression was determined by an immunoblot assay using the indicated antibodies. Data represent averages from triplicate experiments. *, *P* < 0.05; **, *P* < 0.01; ***, *P* < 0.001.

The spike glycoprotein of SARS-CoV-2 binds to the cellular ACE2 receptor, and therefore, it plays a pivotal role in SARS-CoV-2 infection. To further precisely determine the inhibitory step of amuvatinib on the SARS-CoV-2 life cycle, we performed a viral infection assay using pseudotyped virus with S protein (SARS-CoV-2pp) carrying a luciferase gene. Either Vero E6 cells or Calu-3 cells were infected with SARS-CoV-2pp in the absence or presence of various concentrations of either amuvatinib or imatinib, and then viral infection was determined by luciferase activity. Figure 6A shows that amuvatinib significantly decreased SARS-CoV-2pp infection in Vero E6 cells in a dose-dependent manner. Interestingly, imatinib also significantly decreased SARS-CoV-2pp infection in Vero E6 cells (Fig. 6B). We further showed that SARS-CoV-2pp infection was significantly decreased by amuvatinib in Calu-3 cells (Fig. 6C). Meanwhile, imatinib displayed no effect on SARS-CoV-2pp infection in Calu-3 cells (Fig. 6D). Using HEK293T cells cotransfected with both ACE2 and TMPRSS2 expression plasmid, we evaluated the effects of amuvatinib or imatinib on SARS-CoV-2pp infection. As shown in Fig. 6E, SARS-CoV-2pp infection was significantly decreased by amuvatinib in a dose-dependent manner. Notably, imatinib displayed no effect on SARS-CoV-2pp infection (Fig. 6F). In summary, these data indicate that amuvatinib specifically inhibits SARS-CoV-2pp infection in multiple cell lines.

### Amuvatinib displays a broad-spectrum antiviral activity against SARS-CoV-2 variants.

**FIG 7:**
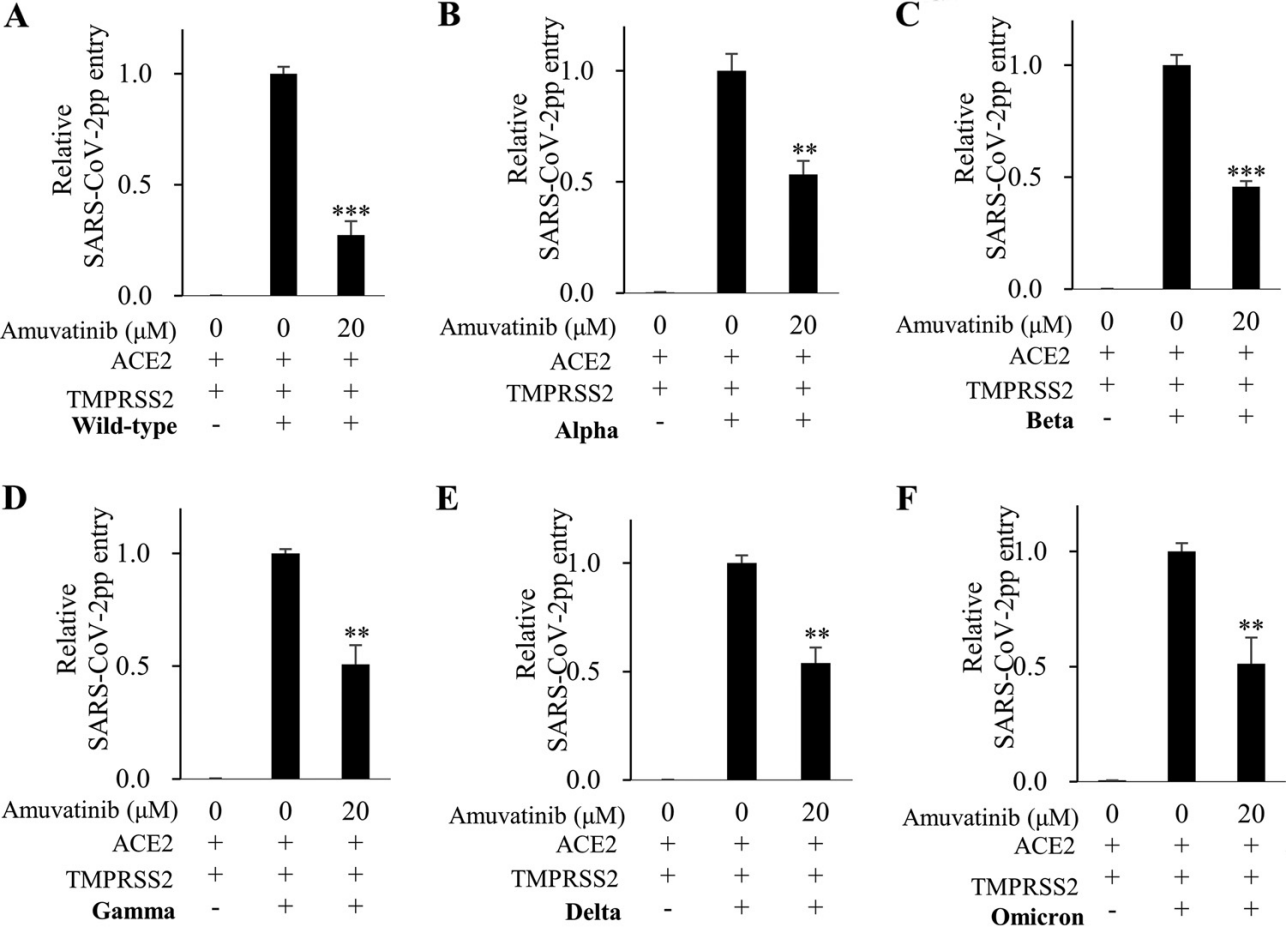
Amuvatinib displays broad-spectrum antiviral activity against SARS-CoV-2 variants. (A) HEK293T cells were cotransfected with ACE2 and TMPRSS2 for 20 h and then treated with either DMSO or 20 μM amuvatinib for 10 min. Cells were infected with wild-type SARS-CoV-2pp in the absence or presence of 20 μM amuvatinib for 1 h, and then culture media were replaced with fresh media without amuvatinib. At 36 h postinfection, the cells were harvested, and viral infection was determined by luciferase assay. (B to F) HEK293T cells were treated as described in panel A using Alpha (B), Beta (C), Gamma (D), Delta (E), and Omicron (F) variants of SARS-CoV-2pp. All experiments were performed in triplicate, and data represent averages. **, *P* < 0.01; ***, *P* < 0.001; Wild-type, Wuhan-Hu-1 (2019-nCoV).

To further investigate the effect of amuvatinib on viral infection in emerging SARS-CoV-2 variants, we generated multiple variants of SARS-CoV-2pp using spike protein. HEK293T cells cotransfected with ACE2 and TMPRSS2 were treated with 20 μM amuvatinib for 10 min and then infected with SARS-CoV-2pp variants. We confirmed that wild-type SARS-CoV-2pp infection was significantly decreased by amuvatinib (Fig. 7A). Consistently, amuvatinib also significantly inhibited viral infection of multiple SARS-CoV-2 variants, including Alpha, Beta, Gamma, Delta, and Omicron (Fig. 7B to F). Although the inhibitory activity of amuvatinib in viral infection of SARS-CoV-2 variants seems to be less significant than that of wild type, these data confirm that amuvatinib possesses a broad-spectrum antiviral activity against SARS-CoV-2 variants.

### Amuvatinib inhibits SARS-CoV-2 infection by disrupting ACE2 cleavage.

**FIG 8:**
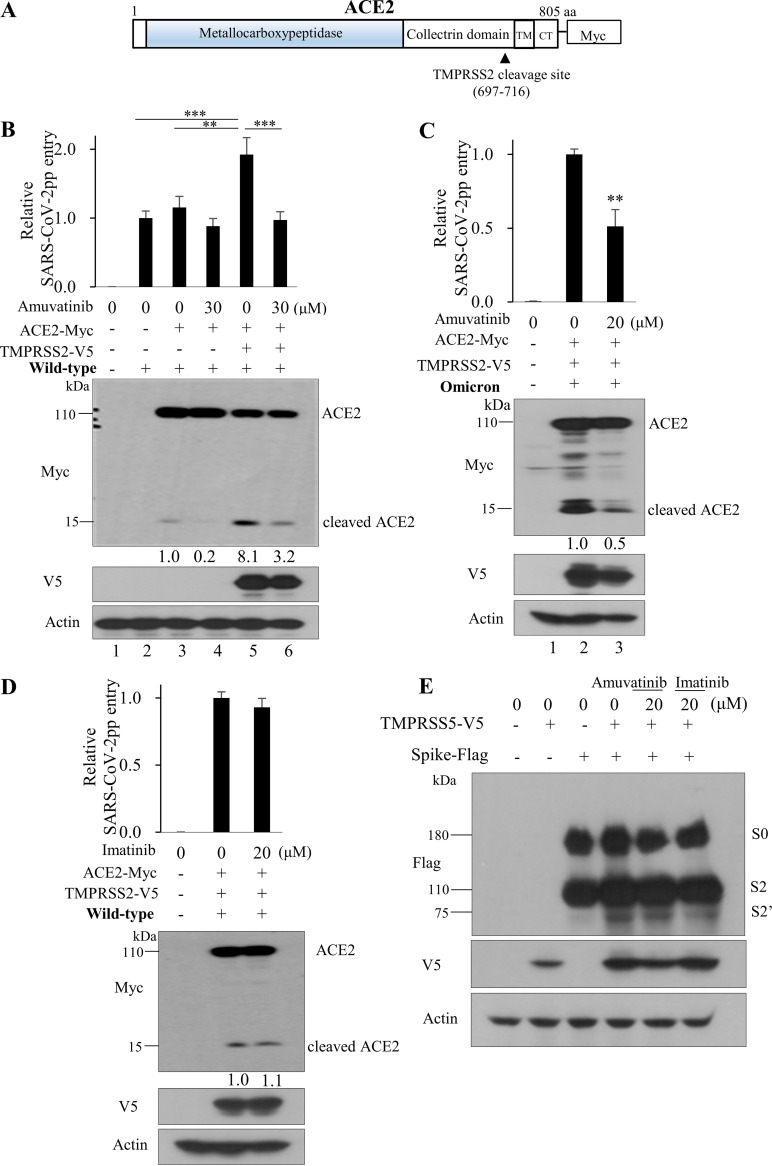
Amuvatinib blocks ACE2 cleavage. (A) Schematic diagram of the domain structure and TMPRSS2 cleavage site in ACE2 protein. (B) HEK293T cells were either transfected with empty vector or Myc-tagged ACE2 or cotransfected with Myc-tagged ACE2 and V5-tagged TMPRSS2 for 20 h. Cells were then treated with either DMSO or 30 μM amuvatinib for 10 min and then infected with wild-type SARS-CoV-2pp for 1 h. At 4 h postinfection, viral infection was determined by luciferase assay. Protein expression was determined by an immunoblot assay using the indicated antibodies. Data represent averages from triplicate experiments. **, *P* < 0.01; ***, *P* < 0.001. (C) HEK293T cells were either transfected with empty vector or cotransfected with Myc-tagged ACE2 and V5-tagged TMPRSS2 for 20 h, treated with either DMSO or 20 μM amuvatinib for 10 min, and then infected with the Omicron variant of SARS-CoV-2pp for 1 h. At 4 h postinfection, viral infection and protein expression were determined as described above. (D) HEK293T cells were treated as described in panel C using imatinib instead of amuvatinib. Viral infection and protein expression were determined as described above. Relative ACE2 cleavage was normalized to β-actin. Protein density was determined using the ImageJ gel densitometry analysis. (E) HEK293T cells were either transfected with empty vector, V5-tagged TMPRSS2, or Flag-tagged spike (S) or cotransfected with V5-tagged TMPRSS2 and Flag-tagged S. At 20 h after transfection, cells were treated with either DMSO, 20 μM amuvatinib, or 20 μM imatinib. At 4 h after treatment, protein expression levels were determined by an immunoblot assay using the indicated antibodies. Immunoblot analysis was performed in triplicate.

SARS-CoV-2 infects host cells by using the viral S protein and cellular ACE2 receptor. The ACE2 is a type I integral membrane glycoprotein 805 amino acids long. TMPRSS2 cleaves the transmembrane C-terminal domain (residues 697 to 716) of ACE2 (Fig. 8A). Overexpression of TMPRSS2 in cells stably expressing ACE2 cleaves ACE2 into two major proteins of 110 kDa and a C-terminal fragment of 15 kDa. Since cleavage of ACE plays an important role in SARS-CoV-2 infection (11), we explored the possible involvement of amuvatinib in ACE2 cleavage. Overexpression of ACE2 minimally increased SARS-CoV-2pp infection. As expected, ACE2 is normally cleaved in wild-type SARS-CoV-2pp-infected cells, and its cleavage is decreased by amuvatinib (Fig. 8B, lane 3 versus lane 4). We confirmed that coexpression of ACE2 and TMPRSS2 markedly increased wild-type SARS-CoV-2pp infection, and this was significantly decreased by amuvatinib. Consistently, the cleaved protein level of ACE2 was markedly decreased by amuvatinib, as determined by densitometric analysis (Fig. 8B, lane 5 versus lane 6). To further determine whether amuvatinib exerts an antiviral activity against SARS-CoV-2 variants, HEK293T cells cotransfected with Myc-tagged ACE2 and V5-tagged TMPRSS2 were treated with amuvatinib and then infected with the Omicron variant of SARS-CoV-2pp. Figure 8C shows that amuvatinib significantly decreased viral infection of the Omicron variant. Consistently, the cleaved protein level of ACE2 was markedly decreased by amuvatinib (Fig. 8, lane 2 versus lane 3). Since imatinib is also a potent inhibitor of RTKs, we asked whether imatinib could inhibit ACE2 cleavage. For this purpose, HEK293T cells were treated as described in Fig. 8C using imatinib instead of amuvatinib, and then the cleaved ACE2 level was determined. As shown in Fig. 8D, imatinib displays no effect on ACE cleavage, suggesting that amuvatinib blocks SARS-CoV-2 infection by specifically inhibiting ACE2 cleavage. We also wondered whether these inhibitors could affect spike protein processing. For this purpose, HEK293T cells were transfected with the indicated plasmids for 20 h and then treated with either dimethyl sulfoxide (DMSO), 20 μM amuvatinib, or 20 μM imatinib. At 4 h after drug treatment, protein expression levels were determined by immunoblot analysis. As shown in Fig. 8E, neither amuvatinib nor imatinib affected processing of the S protein of SARS-CoV-2. In summary, these data indicate that amuvatinib inhibits SARS-CoV-2 propagation at the infection step of the viral life cycle by blocking ACE2 cleavage.

## DISCUSSION

Vaccination is the most effective way to prevent COVID-19. Various types of SARS-CoV-2 vaccines have been developed and administered worldwide. However, due to the emerging variants of SARS-CoV-2, vaccine breakthrough infections are common, and thus, the effectiveness of existing vaccines is being reduced. Moreover, immunocompromised patients are not fully protected by current vaccination regimens (30). Therefore, more effective strategies are needed to control the SARS-CoV-2 pandemic.

As for the therapeutic agents, several SARS-CoV-2 antiviral drugs are being used to treat COVID-19 patients. Both remdesivir and molnupiravir target RNA-dependent RNA polymerase and inhibit viral replication, although the mechanism of action and antiviral activity are different (31). Paxlovid inhibits the viral protease of SARS-CoV-2 and is used for preventing severe cases of COVID-19 in high-risk patients (32). Since hepatitis C virus infection has been successfully controlled by targeting multiple viral proteins (33), it may be also imperative to develop new antivirals aimed at different targets of SARS-CoV-2. As an alternative strategy for antiviral drug development, potential drug candidates may include inhibitors of host proteins because viruses hijack cellular machinery for their own replication. Host-targeting antivirals (HTAs) have a high barrier to resistance and lead to therapeutics with broad-spectrum activity.

Tyrosine kinase plays an important role in the cell signaling system. Drugs targeting tyrosine kinase signaling are currently used in clinical trials for the treatment of cancer (34). It has been reported that tyrosine kinase inhibitors block cancer cell proliferation in the lungs (35). Tyrosine kinase has also been implicated in virus entry and replication (23), and thus, any inhibitors that block tyrosine kinase activity could potentially be drug candidates against SARS-CoV-2 infection. Since SARS-CoV-2 infects human lungs through the respiratory tract, we evaluated the effect of amuvatinib, one of the FDA-approved tyrosine kinase inhibitors, on SARS-CoV-2 infection. A previous chemical library screen study identifies amuvatinib as one of 53 compounds that display antiviral activity against coronaviruses, including hCoV 229E and OC43, but not SARS-CoV-2 (36). In the present study, we found that amuvatinib efficiently blocked SARS-CoV-2 replication with no cytopathic effect in Vero E6 cells. Previous study shows that imatinib, another tyrosine kinase inhibitor, blocks SARS-CoV-2 infection by reducing the expression of ACE2 (37). Here, we showed that not only imatinib, but also amuvatinib, displayed antiviral activity against SARS-CoV-2 infection. Moreover, amuvatinib displayed higher antiviral activity than imatinib against SARS-CoV-2 in both Vero E6 and Calu-3 cell lines, whereas imatinib displayed no anti-SARS-CoV-2 activity in HEK293T cells. In fact, recent studies report that imatinib is not an antiviral drug against SARS-CoV-2 (38, 39). Using SARS-CoV-2pp luciferase reporter assay, we further verified that amuvatinib, but not imatinib, specifically inhibited SARS-CoV-2 propagation at the early infection step of the viral life cycle. Importantly, we demonstrated that amuvatinib specifically inhibited SARS-CoV-2 infection at the binding-attachment step, not the entry-fusion step, of the viral life cycle. Our finding suggests that amuvatinib may represent a potent therapeutic agent for COVID-19.

The cell surface receptor ACE2 can be shed by two cellular serine proteases, ADAM17 (a disintegrin and metalloproteinase 17) and TMPRSS2. TMPRSS2-cleaved sACE2 allows SARS-CoV-2 cell entry, whereas ADAM17-cleaved ACE2 protects organs from inflammatory injuries and regulates intestinal functions (7). Indeed, a previous study shows that ADAM17 is not required for cell entry of SARS-CoV and SARS-CoV-2 (40). The SARS-CoV-2 spike protein binds to ACE2 and then is proteolytically cleaved by TMPRSS2 to trigger the fusion of the viral envelope with the host cell membrane and to facilitate virus entry (3–5). In the present study, SARS-CoV-2pp infection was increased by overexpression of ACE and significantly increased by coexpression of ACE2 and TMPRSS2. Importantly, amuvatinib inhibited ACE- and TMPRSS2-mediated SARS-CoV-2pp infection in a dose-dependent manner. This result suggests that amuvatinib may inhibit SARS-CoV-2 infection by downregulation of intrinsic functions of ACE2 and TMRPSS2. It has been previously reported that tyrosine kinase receptor UFO (AXL) specifically interacts with the N-terminal domain of the SARS-CoV-2 spike protein, and AXL functions as a candidate receptor for SARS-CoV-2 (41). Moreover, AXL promotes SARS-CoV-2 infection in pulmonary and bronchial epithelial cells (41). Since amuvatinib is an AXL inhibitor, it may block SARS-CoV-2 entry by inhibiting AXL activity. To investigate the mechanism of antiviral activity of amuvatinib against SARS-CoV-2, we explored the possible involvement of amuvatinib in ACE2 cleavage. Here, we show that amuvatinib inhibits ACE cleavage, and hence, the C-terminally cleaved ACE2 protein level is decreased in wild-type SARS-CoV-2-infected cells. We further verified that amuvatinib significantly decreased cellular entry of the Omicron variant. It is noteworthy that amuvatinib displays no effect on the processing of the spike protein. This may suggest that amuvatinib inhibits SARS-CoV-2 propagation by inhibiting ACE2 shedding, and thus, viral infection is prohibited. Nonetheless, it is intriguing how amuvatinib inhibits soluble ACE2 levels in SARS-CoV-2-infected cells. Further studies are needed to discover the antiviral mechanism of amuvatinib in the cell entry process of SARS-CoV-2.

A previous study shows that imatinib inhibits SARS-CoV-2 in a luciferase-based pseudoparticle entry assay in Vero E6 cells (37). Moreover, chronic myeloid leukemia patients treated with imatinib show low numbers of COVID-19 cases (42). One randomized clinical trial performed in Spain shows that imatinib might confer a clinical benefit in hospitalized COVID-19 patients (43). On the other hand, our work showed that imatinib inhibited SARS-CoV-2pp entry only in Vero E6 cells at high dosage levels (Fig. 6B), whereas imatinib displayed no effect on SARS-CoV-2pp entry in either Calu-3 (Fig. 6D) or HEK293T (Fig. 6F) cells. This suggests that the effect of imatinib on viral entry differs by cell type. Nevertheless, the results of our study further provided evidence that amuvatinib displays pan-antiviral activity against SARS-CoV-2 variants, including Alpha, Beta, Gamma, Delta, and Omicron. Since the SARS-CoV-2 spike gene is continuously evolving and generating new variants, further studies are needed to evaluate the effects of amuvatinib on emerging SARS-CoV-2 viruses. Taken together, we show that amuvatinib blocks SARS-CoV-2 infection. It is, therefore, worthwhile to develop amuvatinib either as a new drug candidate to treat COVID-19 or as a drug candidate for combination therapy to overcome viral resistance.

## MATERIALS AND METHODS

### Cell culture.

All cell lines, including Vero E6, Calu-3, and HEK293T, were grown in Dulbecco’s modified Eagle’s medium (DMEM) supplemented with 10% fetal bovine serum (FBS) and 1% penicillin-streptomycin with 5% CO_2_ at 37°C as reported previously (44).

### Preparation of infectious SARS-CoV-2.

SARS-CoV-2 (NCCP-43331), SARS-CoV-2 Delta (NCCP-43405), and SARS-CoV-2 Omicron stealth (NCCP-43412) were provided by the National Culture Collection for Pathogens, South Korea. Viruses were cultured in Vero E6 cells grown in DMEM supplemented with 2% FBS, 1% penicillin-streptomycin, and HEPES (Invitrogen, USA). Viral titers were determined by the 50% tissue culture infectious dose (TCID_50_) assay. All experiments were performed in a biosafety level 3 (BSL-3) facility at the Korea Zoonosis Research Institute, Jeonbuk National University (29).

### Production of SARS-CoV-2 pseudoparticles.

Approximately 2.5 × 10^6^ HEK293T cells/well were seeded on 10-cm culture plates and cultured overnight. Cells were transfected with 1 μg of SARS-CoV-2 spike expression plasmid, 3.1 μg of Gag-Pol packaging plasmid, and 3.1 μg of transfer vector encoding the firefly luciferase reporter protein using polyethyleneimine to create wild-type (https://www.addgene.org/149539/), Alpha (https://www.addgene.org/170451), Beta (https://www.addgene.org/170449), Gamma (https://www.addgene.org/170450), Delta (https://www.addgene.org/172320), and Omicron (https://www.addgene.org/180375) variants of SARS-CoV-2pp. At 48 h after transfection, supernatants containing SARS-CoV-2pp were collected and centrifuged at 1,000 rpm for 5 min to concentrate the virus and used for luciferase reporter assay.

### Luciferase assay.

We seeded 4 × 10^5^ HEK293T cells, 4 × 10^5^ Vero E6 cells, and 6 × 10^5^ Calu-3 cells per well on 6-well plates. At 20 h after seeding, HEK293T cells were either transfected with 1 μg ACE2 or 1 μg TMPRSS2 or cotransfected with both plasmids for overexpression. At 24 h after transfection, cells were either left untreated or pretreated with amuvatinib for 10 min and then infected with SARS-CoV-2pp for 1 h at 37°C in the absence or presence of amuvatinib. Cell culture medium was replaced with fresh medium in the absence of amuvatinib. At 36 h postinfection, cells were harvested, and then luciferase assay was performed using the Bio-Glo luciferase assay system (Promega, USA).

### Quantification of RNA.

cDNA was synthesized from total cellular RNAs isolated from Vero E6 cells using a cDNA synthesis kit (Toyobo, Japan) according to the manufacturer’s instructions. Quantitative real-time PCR (qRT-PCR) was performed using the CFX Connect real-time system (Bio-Rad Laboratories) with the following primers: sense, 5′-GTG AAA TGG TCA TGT GTG GCG G-3′, and antisense, 5′-CAA ATG TTA AAA ACA CTA TTA GCA TA-3′ for SARS-CoV-2 polymerase and sense, 5′-TGA CAG CAG TCG GTT GGA GCG-3′, and antisense, 5′-GAC TTC CTG TAA CAA CGC ATC TCA TA-3′, for actin.

### Immunoblot analysis.

Cells were lysed in a cell culture lysis reagent (Promega). Immunoblot assays were performed as described previously (45). The SARS-CoV-2 nucleoprotein was detected using an anti-nucleoprotein antibody (Sino Biological, China). Actin antibody was purchased from Sigma-Aldrich (USA). All immunoblot analyses were performed in triplicates.

### TCID_50_ assay.

The 50% tissue culture infective dose (TCID_50_) assay was performed to determine the infectious titer of cultured SARS-CoV-2. Vero E6 cells were seeded on 96-well plates for 20 h and then infected with 10-fold serial dilutions of the SARS-CoV-2-containing supernatants. At 5 days postinfection, the number of SARS-CoV-2-infected cells was counted under a microscope, and then the TCID_50_ value per milliliter was determined, as previously reported (46). EC_50_ and 50% cytotoxic concentration (CC_50_) values were determined by Prism 9. The selective index (SI) equals CC_50_/EC_50_.

### Water-soluble tetrazolium salt assay.

Vero E6 cells seeded on a 96-well plate were treated with either amuvatinib (Selleckchem, USA) or imatinib (Axon Medchem, Netherlands). At the indicated time points, cell viability was measured using 30 μL of water-soluble tetrazolium salt (WST; Dail Lab, South Korea) as reported previously (47).

### Statistical analysis.

Data are presented as the means ± standard deviations (SDs). Statistical analysis was performed by Student's *t* test for two treatments and one-way or two-way analysis of variance (ANOVA) for multiple treatments. The asterisks in the figures indicate significant differences (*, *P* < 0.05; **, *P* < 0.01; and ***, *P* < 0.001).
