# Supplementary material for: Amuvatinib Blocks SARS-CoV-2 Infection at the Entry Step of the Viral Life Cycle
Source: Microbiol Spectr. 2023 Mar 30;11(3):e05105-22. doi: 10.1128/spectrum.05105-22 (PMC10269473; doi:10.1128/spectrum.05105-22)
Supplement: Supplemental file 1 — Fig. S1 to S4. Download spectrum.05105-22-s0001.pdf, PDF file, 0.2 MB [file spectrum.05105-22-s0001.pdf]

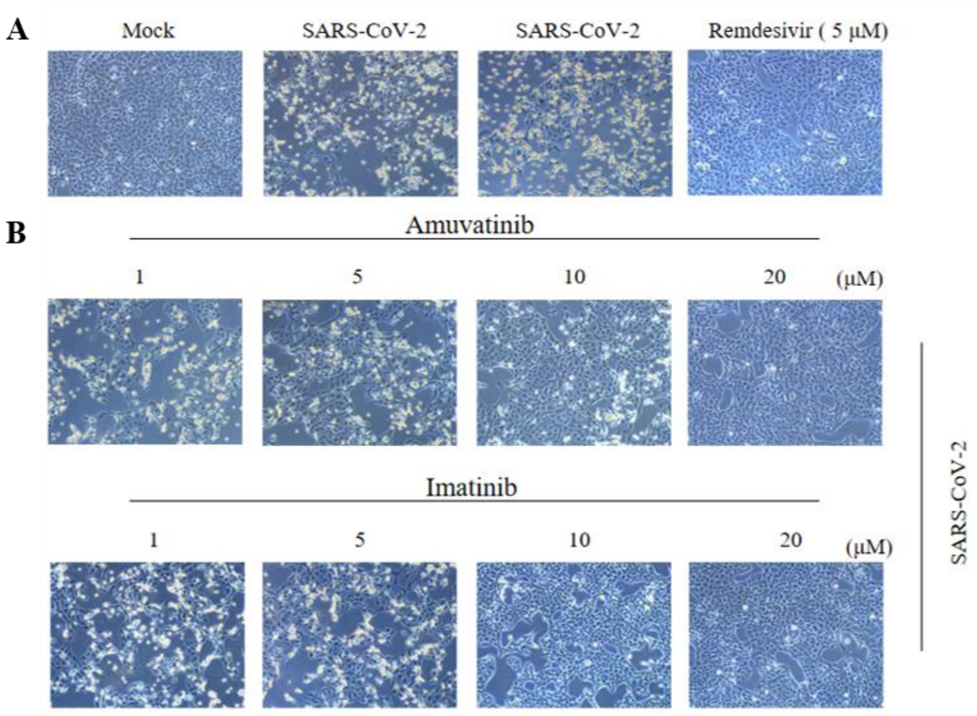

Supplementary Fig 1. Amuvatinib inhibits SARS-CoV-2 infection. (A) Vero E6 cells were either mock-infected or infected with SARS-CoV-2 (MOI = 0.01). At day 2 postinfection, the CPE of Vero E6 cells was visualized under a light microscope. Remdesivir was used as a positive control. (B) Vero E6 cells were infected with SARS-CoV-2 (MOI = 0.01) for 1 h with the indicated concentrations of either amuvatinib or imatinib. Virus-infected cells were further cultured in media containing each drug. At day 2 postinfection, the CPE induced by SARS-CoV-2 in Vero E6 cells was determined under a light microscope. All experiments were performed in a BSL-3 facility.

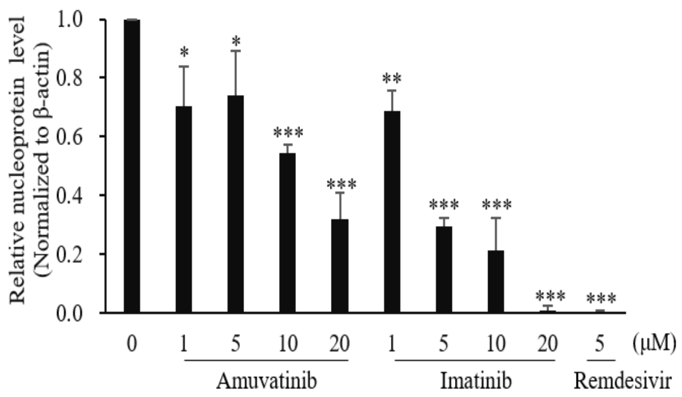

Supplementary Fig 2. Vero E6 cells were either mock-infected or infected with SARS-CoV-2 (MOI = 0.01) for 1 h in the absence or presence of the indicated chemicals and further cultured in media containing various concentrations of either amuvatinib or imatinib. At day 2 postinfection, SARS-CoV-2 protein levels were determined by an immunoblot analysis. Data from triplicate immunoblots are shown as a plotted graph. *P < 0.05, **P < 0.01, ***P < 0.001.

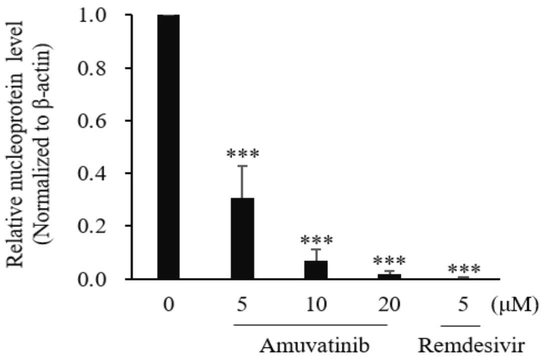

Supplementary Fig 3. Vero E6 cells were either mock-infected or infected with SARS-CoV-2 (MOI = 0.01) in the absence or presence of various concentrations of amuvatinib for 1 h. The SARS-CoV-2-infected cells were further cultured in fresh media containing amuvatinib. At 24 h postinfection, SARS-CoV-2 nucleoprotein levels were determined by an immunoblot assay. Data from triplicate immunoblots are shown as a plotted graph. ***P < 0.001.

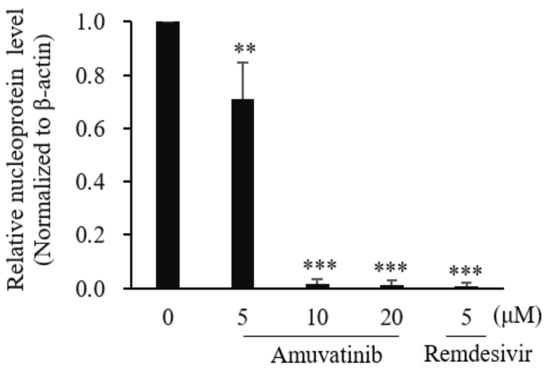

Supplementary Fig 4. Calu-3 cells were either mock-infected or infected with SARS-CoV-2 (MOI = 0.1) for 1 h in the absence or presence of various concentrations of amuvatinib and further cultured in fresh media containing amuvatinib. At 48 h postinfection, cells were harvested to determine protein levels by an immunoblot assay. Data from triplicate immunoblots are shown as a plotted graph. **P < 0.01, ***P < 0.001.
